# Psoriatic Disease and Tuberculosis Nowadays

**DOI:** 10.1155/2012/747204

**Published:** 2012-05-08

**Authors:** Nicola Balato, Luisa Di Costanzo, Fabio Ayala, Anna Balato, Alessandro Sanduzzi, Marialuisa Bocchino

**Affiliations:** ^1^Department of Dermatology, University of Naples Federico II, Via Pansini 5, 80131 Naples, Italy; ^2^Division of Respiratory Medicine, Department of Clinical and Experimental Medicine, University of Naples Federico II, Monaldi Hospital, 80131 Naples, Italy

## Abstract

Psoriasis is a chronic, relapsing and remitting inflammatory skin and joint disease that has a prevalence of 2-3% in the world's population, whereas of 1–2% in Europe. The traditional concept of psoriasis as the “healthy people's” disease has been recently revised because of ever-increasing reports of associations with various pathological conditions (hypertension, Crohn's disease, type II diabetes mellitus, obesity, dyslipidemia, metabolic syndrome, infectious conditions). Particularly, advances in psoriasis therapies have introduced biologic agents. All the tumor necrosis factor-alpha inhibitors are associated with an increased risk of developing active disease in patients with latent tuberculosis infection, because of TNF-**α** key role against *Mycobacterium tuberculosis*. For this reason, exclusion of active tuberculosis and treatment of latent tuberculosis infection are clinical imperatives prior to starting this therapy. Moreover active surveillance for a history of untreated or partially treated tuberculosis or latent form has already been shown to be effective in reducing the number of incident tuberculosis cases.

## 1. Introuction

Psoriasis is a chronic, relapsing and remitting inflammatory skin and joint disease that has a prevalence of 2-3% in the world's population [1], whereas of 1-2% in Europe [2]. In Italy, the number of patients affected with psoriasis is estimated to be greater than 1.7 millions [3]. Men and women are equally affected and exhibit a bimodal distribution with a peak between 15 and 30 years of age and another between 50 and 60 years of age [1]. Psoriasis results from the interaction between genetic and environmental factors [4] and can cause significant impairment of physical, emotional, and psychosocial well-being of patients [5]. The traditional concept of psoriasis as the “healthy people's” disease has been recently revised [5] because of ever-increasing reports of associations with various pathological conditions, such as systemic diseases including hypertension, Crohn's disease, type II diabetes mellitus, obesity, dyslipidemia, metabolic syndrome, and infectious conditions [6]. Furthermore, psoriasis can also lead to substantial adverse socioeconomic consequences for patients, since physical disability and emotional distress of patients can affect their work functions. For these reasons, psoriasis is associated with lesser work productivity and a greater number of missed work days compared to healthy individuals, incurring substantial indirect costs, and adding to the financial burden of the disease together with the incremental economic burden of treating comorbidities in addition to treating psoriasis compared to treating psoriasis alone [7, 8]. Up to 30% of 70% of psoriatic patients require traditional systemic treatments, such as retinoids, methotrexate, and cyclosporine. Many of them imply long-term toxicity, treatment resistance, and potential drug interactions, so only 25% of psoriatic patients are completely satisfied with their treatment [9]. Advances in psoriasis therapies have introduced biologic agents, whose immune targeting is successful in treating many immune-mediated inflammatory diseases. All the tumor necrosis factor-alpha (TNF-*α*) inhibitors are associated with an increased risk of developing active disease in patients with latent tuberculosis infection (LTBI), because TNF-*α* is a key cytokine in protective host defense against *Mycobacterium tuberculosis *(Mtb) [10, 11]. For this reason, exclusion of active tuberculosis (TB) and treatment of LTBI are, therefore, clinical imperatives prior to starting anti-TNF-*α* therapy and active surveillance for a history of untreated or partially treated TB or LTBI has already been shown to be effective in reducing the number of incident TB cases [12–14].

## 2. Psoriatic Disease Pathogenesis

The pathogenesis of psoriasis includes hyperproliferation as well as aberrant differentiation of keratinocytes, dermal angiogenesis, and inflammation. Dermal infiltration of inflammatory T cells, dendritic cells (DCs), macrophages, and neutrophils represents characteristic features of the disease [2]. Nowadays, the fundamental role played by the immune system in psoriatic disease pathogenesis is quite welldefined. T helper (Th)1 and Th17 lymphocytes contribute to the pathogenesis of psoriasis through the release of inflammatory cytokines that promote further recruitment of immune cells, keratinocyte proliferation, and sustained inflammation [15]. The T lymphocytes involved in lesion development were initially thought to be Th1 differentiated, based on interferon- (IFN-) gamma and interleukin- (IL-) 2 productions. Th17 cells have recently been classified as distinct from Th1 and Th2 subsets. They are defined by the ability to synthesize IL-17 in response to antigen-presenting cell-derived IL-23 and other differentiating cytokines. In addition, Th17 cells have been reported to cosynthesize IL-17 and IFN-gamma as well as IL-22 [16]. Recently, psoriatic skin lesions are reported to have increased gene expression of IL-23, IL-17, and IL-22 [17]. IL-23 is a heterodimeric cytokine composed of two subunits (p40 subunit, common with IL-12, and p19 subunit, specific for IL-23) [18]. IL-23 is produced by dendritic cells (DCs), macrophages, and other antigen-presenting cells under the influence of some Gram-positive as well as Gram-negative bacteria and lipopolysaccharides [18]. Several recent studies suggest that psoriasis is a Th17 cell-mediated disease driven by IL-23 [19]. Moreover, TNF-*α* stimulates CD11^+^ inflammatory DCs to produce IL-23 and IL-20 and appears to be a critical cytokine for many of the clinical features of psoriasis, including keratinocyte hyperproliferation, endothelial cell regulation, and recruitment/effector function of memory T-cells. All these findings reinforce that psoriasis pathogenesis is a complex interaction among genetic, immunological, and environmental components.

## 3. Clinical Phenotype and Histological Psoriasis Features

Clinical diagnosis of psoriasis is relatively easy for a dermatologist, especially when the lesions present as erythematous, sharply demarcated indurated plaques with silvery white scales. Plaques may have an oval or irregular shape, varying from one to several centimetres in diameter and are usually distributed symmetrically on the extensor surfaces of limbs (mainly elbows and knees), the lower back and the scalp. Itching is variable, but it is usually absent [20]. These clinical aspects reflect the histopathological findings observed in active lesions, characterized by hyperkeratosis, parakeratosis, diminution, or loss of the granular cell layer, acanthosis of the epidermal ridges, tortuous and dilated blood vessels, and perivascular leukocytic infiltrate in the dermal papillae [1]. The clinical and histological features of chronic plaque psoriasis are generally sufficient to make the diagnosis. Furthermore, psoriasis can present many faces, including guttata, pustular, and erythrodermic. Guttate psoriasis is characterized by the acute onset of round, erythematous, slightly scaling papules over the trunk and extremities. The face could be involved. The disease is self-limiting; however, a proportion of affected individuals may progress to a more chronic form of plaque psoriasis. Flares of guttate lesions may appear during the course of chronic plaque psoriasis and can follow streptococcal infection (particularly of the upper respiratory tract) and/or acute stressful life events [21]. Generalized pustular psoriasis, as well as the localized form and its variants (circinate or Bloch-Lapière's pattern, acrodermatitis continua of Hallopeau) are characterized by nonfollicular sterile pustules, which represent the macroscopic aspect of the massive neutrophil infiltration of epidermis [21, 22]. The erythrodermic form is dominated by generalized erythema, loss of peculiar clinical features of psoriasis, and skin failure, that is, inability to maintain homeostatic functions [23]. Psoriatic erythroderma is not substantially different from erythroderma by other causes.

## 4. Psoriasis and Metabolic Comorbidities

 It has recently been found that psoriatic patients have a higher prevalence of some metabolic disorders [24], particularly obesity, diabetes, or abnormal glucose intolerance, dyslipidemia, and systemic hypertension, which together are known as the metabolic syndrome [25]. Psoriasis is now also considered to be a marker of increased cardiovascular risk, especially in young patients [26]. Psoriatic disease is associated with unhealthy behaviors, particularly smoking and obesity; in addition, it may influence metabolic and cardiovascular risk independently of lifestyle factors, through common genetic risks, resulting in a chronic systemic inflammatory pathway [27]. A recent study, evaluating the association with comorbidities in psoriasis patients in Italy, showed that, from a total sample of 511, 532 individuals, overall patients had more selected comorbidities compared to healthy controls, in particular chronic ischemic heart disease, obesity, diabetes mellitus, bronchitis, cardiac valve abnormalities, dermatomycosis, benign mammary dysplasias, disorders of penis, disorders of external ear, inflammation of eyelids, and contact dermatitis. In agreement with previous studies, they found a significant association of psoriasis with cardiovascular risk factors (diabetes mellitus and obesity) without, however, confirming an association with others (dyslipidemia and blood hypertension). In contrast, we found no significant difference in general medical history (e.g., cardiac diseases, diabetes) between psoriatic and control groups, except for high blood pressure that was more prevalent in psoriatic patients [28–31].

A causal link between psoriasis and cardiovascular disease is hypothesized also for the involvement of the same mediators and markers of inflammation, mainly TNF-*α*, IL-6, fibrinogen, and C-reactive protein [6]. Apart from these cytokines, insulin-like growth factor (IGF)-I, the main anabolic mediator of somatotroph axis also acting as an autocrine/paracrine signal essential for proliferation of epidermal keratinocytes, has been found to be overproduced in psoriatic epidermis. Despite the increase in IGF-I in psoriatic plaques, psoriatic patients exhibited low circulating levels of IGF-I, with a negative correlation to Psoriasis Area and Severity Index (PASI). However, it is well known that a number of inflammatory cytokines affecting IGF-I secretion and subtle changes in IGF-I levels have been associated with unfavourable lipid profiles, with increased cardiovascular mortality [32]. Thus, although abnormalities in somatotroph axis activity have been hypothesized to account for the low IGF-I levels in the psoriatic patients, with a possible primary or secondary effect of these disturbances on the psoriasis process modulation [33], the more likely association of low IGF-I with the common inflammatory pathways of both metabolic syndrome or psoriasis has not been considered as far. On this basis, Savastano et al. speculated that in psoriasis chronic inflammation might be an important modulator of low IGF-I status and that, similarly to other pathological conditions, low IGF-I status could be added as a further possible mechanistic link between psoriasis and associated metabolic comorbidities [34]. In conclusion, psoriatic systemic inflammation may underneath insulin resistance, which in turn triggers endothelial cell dysfunction, leading to atherosclerosis and finally myocardial infarction or stroke [27].

## 5. Psoriasis and Malignancy

Although anti-TNF-*α* drugs mechanism of action has been well investigated, long-term studies concerning malignancy risk associated with these immunosuppressive agents have been most extensively performed in rheumatoid arthritis more than in psoriasis population; there are in fact just some case reports regarding this matter in psoriasis, suggesting that these therapies can permit malignant processes [35]. Therefore, risk of malignancy with anti-TNF-*α* in psoriasis remains unclear. However, the majority of reports indicate that TNF-*α* inhibitors may cause a slightly increased risk of cancer, including nonmelanoma skin cancer and hematologic malignancies [36, 37]. So far, it is worthy that oncologic personal and familiar history, skin examination, and baseline blood tests attempting to identify any hematologic abnormalities be required before starting biologics therapy [35].

## 6. Psoriatic Infectious Co morbidities (Other than TB)

TNF-*α* plays an important role in host defense and anti-TNF-*α* agents may theoretically increase the risk of infections. Most recent studies suggest that anti-TNF-*α* agents are associated with a slight increased risk of serious infections, especially in the early phase of treatment and an absolute rate of infections relatively low [38]. Grijalva analyzed whether initiation of TNF-*α* antagonists compared with nonbiologic drugs was associated with an increased risk of serious infections in a cohort of patients affected by rheumatoid arthritis, inflammatory bowel disease, psoriasis, psoriatic arthritis, or ankylosing spondylitis; rates were 5.41 for TNF-*α* antagonists and 5.37 for traditional systemic drugs per 100 person-year, showing no significant difference between the 2 groups [39]. Another recent systematic review showed that there may be a small increased risk of overall infection with short-term use of TNF-*α* antagonists in psoriasis, whereas 97.6% were nonserious infections and the large majority of these were ones of the upper respiratory tract [40].

## 7. Psoriasis and TB

Recently, infliximab, etanercept, adalimumab, and golimumab have become the drugs of choice in the treatment of these disorders. Of course, such kinds of drugs could conceptually interfere with a cytokine, TNF-*α*, which is crucial in the development and maintenance of the granuloma. Moreover, the early diagnosis and treatment of individuals harboring the Mtb is key to ensuring the effectiveness of health programs aimed at the elimination of TB. On the other hand, psoriasis *“per se*” could represent an independent risk factor for TB since, interestingly, an unexpected high prevalence was found in patients affected by such a disease (18.0%), even adjusting for age, work, and other parameters [41]. A similar result is reported by Bassukas et al. during a two-year period, LTBI diagnosis rate was compared in consecutive patients with psoriasis or inflammatory bowel disease like Crohn's disease or ulcerative colitis: these patients had significantly smaller tuberculin skin testing compared to psoriasis patients (*P* = 0.007). Applying LTBI diagnosis guidelines, latent infection resulted in more psoriasis (50%) than inflammatory bowel disease patients (24.2%), prior to onset of any anti-TNF-*α* treatment (*P* = 0.04) [42]. A recent survey concerning the evaluation of the infectious complications during biological therapy of psoriasis showed a rate of infections of 12.24%, with only one case of pulmonary TB, out of 988 patients [43]. The authors stressed that such a result depend on a strict screening of LTBI, prior to starting the biological treatment. A French report showed that, in a mixed population of patients treated with TNF-*α* blockers, including psoriasis, 45 cases were collected of non-TB opportunistic infections (OIs). One-third (33%) of OIs were bacterial (4 listeriosis, 4 nocardiosis, 4 atypical mycobacteriosis, 3 nontyphoid salmonellosis), 40% were viral (8 severe herpes zoster, 3 varicella, 3 extensive herpes simplex, 4 disseminated cytomegalovirus infections), 22% were fungal (5 pneumocystosis, 3 invasive aspergillosis, 2 cryptococcosis), and 4% were parasitic (2 leishmaniasis). Ten patients (23%) required admission to the intensive care unit, and four patients (9%) died. Risk factors for OIs were treatment with infliximab (OR = 17.6 (95% CI 4.3–72.9); *P* < 0.0001) or adalimumab (OR = 10.0 (2.3 to 44.4); *P* = 0.002) versus etanercept, and oral steroid use >10 mg/day or intravenous boluses during the previous year (OR = 6.3 (2.0 to 20.0); *P* = 0.002) [44]. Another study identified 69 cases of tubercular active disease prospectively through the French RATIO registry: the sex and age-adjusted TB incidence rate was 1.17 per 1,000 patient-years, 12.2 times that of the general population [45]. A similar conclusion was reached by a Portuguese biologics registry study that found the TB risk with anti-TNF-*α* antibodies to be 12-fold greater than with etanercept [46]. Sánchez-Moya et al. report that, among one hundred and forty-four patients with moderate-to-severe psoriasis treated with anti-TNF-*α* agents, a total of 42 (29%) patients were diagnosed with LTBI based on a positive tuberculin skin test (TST) or re-TST, and/or signs of past TB in the chest X-ray. All of them received chemoprophylaxis with isoniazid (H). Only one patient developed an active lymphnode TB [47]. Besides, the risk of active TB due to infliximab appeared to be twice that of etanercept [48]. Most of the active TB cases in patients treated with TNF antagonists are due to reactivation of latent infection with Mtb. TB in patients who have been treated with TNF antagonist therapies usually progresses rapidly and is frequently disseminated, with several extra-pulmonary localizations. Thus, the most effective way to avoid TB reactivation is (and remains) treatment of the latent infection.

## 8. Management of TB Infection in Psoriasis Patients

### 8.1. Conventional TST and Interferon Gamma Release Assays: Overview on Test Characteristics

Screening for LTBI before the initiation of any immune-suppressive therapy regimens, including TNF-*α* blocking agents, is part of current management strategies of common inflammatory disorders such as psoriasis, such an approach having been associated with an estimated reduction of more than 80% of the risk of TB reactivation [49, 50]. Guidelines for TB prevention suggest taking a careful medical history and excluding active TB before starting any kind of treatment. All patients should be questioned regarding their demographic details (i.e., age and country of birth), history of previous Bacillus Calmette-Guèrin (BCG) vaccination, TB risk factors (i.e., recent close exposure to active TB cases, immigration from or recent stay in high TB prevalence areas, and chest X-ray evidence of TB *sequelae*), and current treatments (i.e., drugs) [51].

Diagnostic algorithms based on the use of the TST are still in use worldwide for the detection of TB infection. As known, TST is a measure of a delayed-type-hypersensitivity response to the intradermal inoculation of PPD (purified protein derivative), which is a mixture of more than 200 mycobacterial antigens. Despite TST is easy, safe, and inexpensive to administer, poor specificity limits its use because of PPD cross-reactivity with environmental mycobacteria and with the *M. bovis *BCG vaccine strains. In addition, despite different cut-off values are used to stratify PPD reactors according to their likelihood to be infected and to predict the risk of disease progression, sensitivity of TST is significantly reduced in the case of immune-compromise where a TST-positivity is given by an induration area equal or greater than 5 mm [51, 52]. Finally, TST result may not be stable over time as the reaction size may increase due to a new infection (conversion) or to serial testing in previously sensitized individuals (boosting). Reversion to a negative TST result may also occur [53]. Overall, these limitations explain why in the guidelines released by the British Thoracic Society (BTS) for the management of patients due to start anti-TNF therapies, the use of TST was not recommended in patients with no TB risk factors, while remaining controversial in high-risk cases due to the expected high rate of false-negative results [54].

Recently, commercially available and FDA (Food and Drug Administration) approved interferon gamma release assays (IGRAs) have been introduced in clinical practice as alternative tools for the identification of Mtb infection. Unlike TST, blood tests are based on the principle of detecting IFN-*γ*  □ production by effector memory T-cells upon short term (16–20 hrs) *in vitro *stimulation with TB-specific antigens. Measurements of IFN-*γ* are performed either by ELISpot-based assay (T-SPOT.TB, TS-TB, provided by Immunotech, UK) or ELISA [QuantiFERON TB Gold (QFT-G) and QFT-in-tube (QFT-IT), both provided by Cellestis, Australia]. All tests rely on the use of two TB specific antigens, that are *early secretory antigen*- (ESAT-) 6 and *culture filtrate protein- *(CFP-) 10, mapped to a genomic region called RD (*region of difference*)-1, which is absent in the vaccine strains and in most non-TB mycobateria (expect * kansasii, szulgai*, * marinum*, * flavescens*, and * gastrii*) [55]. To date, QFT-IT is the latest improvement of the ELISA technology. It has widely replaced the previous in-plate format (QFT-G) as blood samples are directly collected into tubes pre-coated with antigens (also including a third antigen, that is, the RD-11-related TB7.7) and ready for incubation. Each test is provided of both a negative and a positive control (phytohemagglutinin, PHA), thus allowing a more comprehensive evaluation of the host immune reactivity. Despite there is evidence that IGRAs performance (i.e., QFT) may significantly vary in comparison to TST depending on the epidemiological and clinical setting [56, 57], overall, a pooled sensitivity of 87.5% and 81% has been estimated in a recent meta-analysis, respectively, for TS-TB and QFT-IT, as compared to TST (70%). Pooled specificity has instead been evaluated at 86% for TS-TB and 99% for QFT-IT. The pooled estimated rate of indeterminate results was low, 2.1% (95% CI, 0.02–0.023) for QFT-IT and 3.8% (95% CI, 0.035–0.042) for TS-TB, increasing to 4.4% (95% CI, 0.039–0.05) and 6.1% (95% CI, 0.052–0.071), respectively, among immunecompromised hosts [58]. As for TST, a main limitation of IGRAs is represented by the lack of discrimination of LTBI from active TB. Reproducibility in serial testing along with definitions of conversion and reversion, differentiation of cut-off values for targeting selected patient populations, positive predictive value, and boosting remain as further areas of uncertainty that only in part have recently been addressed, as elsewhere discussed [59, 60]. In conclusion, IGRAs testing requires the availability of equipped laboratories with expertise in the field and adequate economic resources to ensure efficient samples turnover. Although intrinsic technical characteristics may explain a certain degree of discrepancy when comparing the performance of the two IGRA formats, current clinical evidence actually does not clearly favor one test over the other in any setting.

### 8.2. TST and Advances on IGRAs Performance in Psoriasis Patients

Nonetheless, some recent evidence still supports that the use of TST is reliable as an effective diagnostic approach for the detection of TB infection, as suggested by Sanchez-Moya et al. in a prospective evaluation of 144 patients affected by moderate-to-severe psoriasis in Spain [47], some concerns on TST application in this selected patient population need to be addressed. TST may be ineffective due to the dubious results that it generates in patients with psoriasis as disease activity may substantially affect the test outcome [60]. First, it may be impossible to find lesion-free skin areas suitable for TST in patients with severe skin disease. Secondly, the observation of an increased TST reactivity of even healthy skin regions has been associated with proinflammatory priming that leads to an over-reaction to a wide spectrum of antigenic triggers [61]. In this issue, Bassukas et al. [42] have recently shown that patients with moderate-to-severe plaque psoriasis had significantly larger TST reactions compared to patients with inflammatory bowel disease, as previously reported. To overcome TST limitations, assessment of the value of IGRAs as diagnostic tools in detecting LTBI in psoriasis patients is currently under investigation, most of the studies being focused on patient candidates for receiving anti-TNF agents due to the high impact of such a therapy on TB risk [62, 63]. Chiang et al. [64] in a recent prospective study first used TS-TB as a unique diagnostic tool for TB infection screening purposes in 63 patients affected by severe psoriasis in UK. The Authors found a prevalence of LTBI of 7.9%, the IGRA test positivity being associated with a travel history to TB endemic countries. A retrospective analysis carried out in Switzerland over a 4-year period on 50 psoriasis patients, the 90% with prior BCG vaccination, subjected to both TST and TS-TB before the initiation of anti-TNF treatment showed that a positive TS-TB result was strongly associated with a presumptive diagnosis of LTBI, while this was not the case for TST. Agreement between the two tests was quite poor (*κ* = 0.33). LTBI treatment was avoided in the 20% of patients tested positive by TST (≥5 mm) but negative by TS-TB (however, one case of disseminated tuberculosis occurred despite LTBI treatment after 28 weeks of therapy with adalimumab) [64]. More recently, a further retrospective study conducted in Taiwan, an intermediate TB burden country, has assessed the accuracy of QFT in 147 psoriasis patients, including both cases with and without treatment with TNF-alpha blocking agents (median exposure of 24 weeks) [65]. Previous survey reports in this setting have reported a TST-positive rate of 47% in the general population aged 20–59 years old [66]. In the study by Chiu et al. the percentages of LTBI in psoriasis patients ranged from 30% to 36%, according to TST cut-off used, that was 10 or 5 mm, respectively, the overall rate of QFT positive tests being even lower (12%). Agreement with TST was poor (63%, *κ* = 0.046). Despite this, unnecessary treatment of LTBI was avoided in a significant number of cases, active TB developed in one out of four QFT-positive patients (25%) that while receiving TNF blocking agents were not treated for LTBI [65]. Finally, a cross-sectional study realized in Brazil has shown that the frequency of TST-positive responses and skin induration size were significantly lower in 33 psoriasis patients (18%; 2.6 ± 0.7 mm) as compared to 30 cases affected by other common dermatological diseases (control group) (53%; 9.3 ± 1.4 mm) [67]. Conversely, frequencies of TS-TB-positive results were not different in psoriasis (47%) and control patients (40%), while a poor agreement with TST was recorded in the formers (*κ* = 0.375). These findings confirm a previous observation of a decrease of central memory anti TB immune responses in untreated psoriasis patients living in endemic areas while they retained T-cell memory effector activity [68]. Overall, rates of indeterminate results were quite low ranging from 1.6% (TS-TB) [63] to 1.9% (QFT-IT) [65]. As indeterminate results may reflect a high background IFN-gamma production (negative control) or, alternatively, the inability of the immune system to mount a T-cell response (positive control), every attempt should be made to clarify the reasons, also excluding technical errors, behind this kind of results [69]. Head-to-head comparison studies of TS-TB versus QFT are even more limited. In this issue, we simultaneously tested a small cohort of patients affected by psoriasis or psoriatic arthritis with TST in comparison with both TS-TB and QFT-IT. The main finding of the study was a good agreement of blood tests with conventional TST (*κ* = 0.86 and 0.84, resp.). This was not surprising due to the high rate of negative results recorded as patients were mainly represented by non-BCG vaccinated young individuals without known TB risk factors, confirming a previous report [70]. However, in two TST-negative cases (11%), IGRAs yielded a positive result that allowed the identification of a presumptive LTBI [71], thus supporting using these assays in psoriasis patients [72]. Finally, only a few studies to date report on the performance of TST or IGRAs for monitoring patients already under treatment with TNF-*α* blockers. Single-case reports and case series have described psoriasis patients tested negative by TST but positive by means of an IGRA [73, 74]. Clinical applicability of QFT-IT has been prospectively assessed in 50 patients with psoriasis along with TST while patients were on anti-TNF therapy. Agreement among tests was moderate (*κ* = 0.408) at baseline, good (*κ* = 0.734) at 6 months, and fair (*κ* = 0.328) at 12 months of treatment [75]. To date, TST+/QFT- cases were regarded as not suffering from LTBI which instead was 12 diagnosed based on QFT conversion alone. Overall, current evidence including disease conditions other than psoriasis suggests IGRAs results to be not interpretable in this setting [76–78]. Longitudinal high-powered studies with longer followup periods are necessary to optimize their use and systematically assess whether IGRAs can be used in this clinical scenario for monitoring a previous infection or for regular screening purposes in high prevalence countries or more specifically after an exposure event in low burden areas in the case of occurrence of a new infection.

### 8.3. National Guidelines and Consensus Recommendations: Points of Agreement and Criticisms

Actually, views on how TST and IGRAs should be employed for LTBI screening are widely divergent and no specific recommendations in the setting of patient candidates for receiving immune-suppressive therapies are available [79]. Among the disease conditions for which LTBI diagnosis is mandatory, psoriasis occupies a unique position due to some specific disease-associated issues. Overall, the present observations suggest that TST-based screening algorithms may lead to over-diagnosis of LTBI in patients affected by overt plaque psoriasis or, conversely, may be not applicable in the case of extensive skin involvement. In addition, a likely impairment of central memory T-cell responses leading to TST unresponsiveness may further represent an issue of concern in these patients, at least in TB endemic areas. This means that innovative approaches are needed to overcome these limitations through the readaptation of current guidelines while suggesting a key role of IGRAs as first-line diagnostics for putative LTBI at least in patients with diffuse skin morbidity and in the case of confounding factors, like previous BCG vaccination. In 2005, the U.S. Centers for Disease Control and Prevention (CDC) first recommended the use of QFT-G (the plate ELISA-based format available at that time) instead of TST in all circumstances and clinical settings in which TST was used [80]. However, this is not the same approach recommended in the last update where different strategies are suggested including testing with both an IGRA (with no test format preference) and a TST in the case of high suspicion of false-negative or false-positive results, indeterminate, borderline, or invalid results [81]. In the European setting in 2006, the National Institute for Clinical Excellence (NICE) guidelines proposed a cost-effective two-step strategy that is confirmation of a positive TST result by any available IGRA [82]. It was, however, suggested to consider IGRAs as an alternative tool in the case of not reliability of TST or of high suspicion of false-negative TST results in immune-compromised patients. Later on, IGRA tests were offered as a suitable alternative to TST in all BCG-vaccinated individuals within the context of this risk assessment by the Heath Protection Agency (HPA) [83]. Despite the NICE guidelines were not focused on the management of patients to be treated with TNF blockers, Lalvani has recently suggested that it may be prudent to perform both TST and any IGRA to maximise diagnostic accuracy until stronger evidence on blood tests use in this patient population has expanded sufficiently [55]. Despite the Canadian and Italian guidelines which are in line with the NICE approach [84, 85], further recommendations by national societies worldwide still have divergent positions when targeting patient candidates to immune-suppressive/anti-TNF therapies. To date, most of them, except those from Switzerland and Germany, recommend TST (mainly as a one-step strategy) as the unique screening tool, with cut-off values ranging from 5 to 10 mm in the different geographic settings. Conversely, any commercial IGRA is preferred instead of TST in Switzerland, while Germany guidelines recommend the combined use of TST (cut off ≥5 mm) only in IGRA-negative cases with clinically proven TB exposure [45, 50, 86–93]. The more recent TBNET consensus statement [48], based on published evidence and expert opinions, suggests using any IGRA, or, as an alternative, the TST testing one-step strategy (cut off ≥10 mm) in patients with no prior BCG vaccination. Repeated testing for TB infection may be considered in patients with ongoing risks of exposure. In this case, the use of TST is not strictly recommended due to the boosting effect. Overall, in all the scenarios proposed, no differences among disease conditions to be targeted are considered and no specific recommendations are proposed to tailor specific disease phenotypes, such as severe skin psoriasis. In this issue, the unique document specifically focused on psoriasis patients provided by the National Psoriasis Foundation suggests the use of TST as first-line screening tool (cut-off equal or greater than 5 mm) and considering IGRA testing in BCG-vaccinated patients. However, there is no mention of specific disease subtypes [87].

### 8.4. Treatments Options for LTBI and Active TB

As recently summarized in the TBNET consensus statement, recommended treatment regimens for LTBI vary, their efficacy having not been evaluated in this setting. They mainly include 6 or 9 months with H, 3 months of rifampicin (R) plus H, and 4 months of R [48]. The time delay before starting anti-TNF agents also differs ranging from 3 weeks to as long as possible after the initiation of TB prophylaxis, depending on the geographical context [45, 50, 86–93]. Strict adherence to treatment has to be strongly encouraged as it significantly reduces the patient risk to develop active TB. Patients should be educated about signs and symptoms of possible TB reactivation or of drug-induced side effects. Baseline and routine followup of liver enzymes should be performed on a monthly basis. Repeated chest X-ray is not recommended. Imaging of the thorax also including high-resolution-computed tomography scan should instead be performed without delay in the case of suspected active TB. No action is required for patients having completed a previous course of anti-TB treatment unless a reinfection is plausible. Treatment regimens are recommended in the case of active TB according to international standards as for susceptible immune-competent patients [94]. No differences in treatment duration have been suggested as actually there is no evidence for the need of any prolongation. The optimal timing for the initiation of TNF-blocking therapies is unclear, in some instances being recommended after the completion of at least 2 months of anti-TB treatment. Expert opinion in agreement with the CDC conversely suggests waiting until the end of a full course. Maintaining vigilance for TB even after the completion of appropriate treatment strategies (LTBI/active TB) remains of utmost clinical relevance.

## Abbreviations

TNF-*α*: Tumor Necrosis Factor-*α*
TB: Tuberculosis
Mtb: *Mycobacterium tuberculosis*
LTBI: Latent tuberculosis infection
DCs: Dendritic cells
Th: T helper
IFN-gamma: Interferon-gamma
IGF-I: Insulin-like growth factor-I
PASI: Psoriasis Area and Severity Index
OIs: Opportunistic infections
TST: Tuberculin skin test
H: Isoniazid
R: Rifampicin
BCG: Bacillus Calmette-Guèrin
PPD: Purified protein derivative
BTS: British Thoracic Society
FDA: Food and Drug Administration
IGRAs: Interferon gamma release assays
T-SPOT.TB, TS-TB: ELISpot-based assay
QFT-G: ELISA QuantiFERON TB Gold
QFT-IT: QFT-in-tube
ESAT-6: Early secretory antigen-6
CFP-10: Culture filtrate protein-10
RD-1: Region of difference-1
PHA: Phytohemagglutinin
CDC: Disease control and prevention
NICE: National Institute for Clinical Excellence
HPA: Heath Protection Agency.
